# Supplementary material for: Factors associated with the uptake of intermittent preventive treatment for malaria during pregnancy in Cameroon: An analysis of data from the 2018 Cameroon Demographic and Health Survey
Source: PLOS Glob Public Health. 2024 Mar 27;4(3):e0001245. doi: 10.1371/journal.pgph.0001245 (PMC10971583; doi:10.1371/journal.pgph.0001245)
Supplement: Attachment — Submitted filename: Response to reviewers.pdf [file pgph.0001245.s002.pdf]

March 13th 2023

Guimsop Dominique Ken
University of Dschang
BP 67, Dschang Cameroon
dk.guimsop@outlook.com
(+237) 698684550

To,

Dr Catherine Kyobutungi
Editor-in-Chief
PLOS Global Public Health

Subject: A rebuttal letter

Dear Dr Kyobutungi,

I am pleased to submit the revised version of my original manuscript after consideration of the how pertinent comments of the reviewers. I wish to thank you and the team for the meticulous work, and hope to hear from you soon.

You shall find below under the heading "response to reviewer" an answer to each point raised by the editor and reviewer(s). Each comment is first stated, followed by a paragraph beginning with an "A:" standing for "Answer:".

Response to reviewers

- **Reviewer #3:**

General

1. Title: 'Coverage and factor associated to the uptake of Intermittent preventive treatment for malaria during pregnancy (IPTp) in Cameroun in 2018: an analysis from the 2018 Cameroon Demographic Health Survey data' could be revised to 'Factors associated with the uptake of Intermittent preventive treatment for malaria during pregnancy (IPTp) in Cameroun in 2018: an analysis from the 2018 Cameroon Demographic Health Survey data' as Coverage is implied in the 'uptake'.

A: Alright, the title was revised accordingly.

2. Generally, it would be good to have a fluently speaking English health personnel edit the manuscript for general improvement in the expression of the language in the manuscript as there are some few errors in some of the sentences. For example, the first sentence of the Abstract in lines 20-21 'Malaria in pregnancy is a major public health issue, contributing to a

significant increase in pregnant women and children mortality and morbidity' could read as follows: 'Malaria in pregnancy is a major public health issue, contributing to a significant increase in maternal and child mortality and morbidity'. And in lines 27-28, 'Data were collected using a questionnaire administered in face to face to mothers with at least one child under 5, selected using a 2-stage stratified sampling process.' Could read 'Data were collected using a questionnaire administered face to face to mothers with at least one child under 5, selected using a 2-stage stratified sampling process.' I recommend a thorough reading through the manuscript to correct minor typographical and grammatical errors.

A: Yes, we had the manuscript read again by ourselves and by a fluently speaking English health personnel.

Abstract

3. Authors mention in line 30 that 'a case-control design was used to assess…'. Could the study design be an analytical cross-sectional design instead? Demographic and Health surveys are cross-sectional studies.

A: Yes, we removed that part

Introduction

4. Line 64: 'once month apart' should read 'one month apart'

A: You are right, corrected.

5. Line 71: 'u' is missing from 'sing' to read 'using' chloroquine… Line 78, antenatal is spelt wrongly at the beginning of the sentence, t is also missing from 'that'.

A: corrected.

6. Line 80: poor uptake has been referred to as IPTp-SP $\geq$ 3 doses. This should be corrected.

A: Corrected, thank you.

Study population

7. Do Authors mean biological mothers of children less than five years who were between the ages of 15-49 years? The statement in lines 101 – 103 is not clear.

A: Yes, that is what we meant. we reformulated the sentence.

Data management

8. Data grooming is not the same as data cleaning. Did Authors mean data cleaning in line 126?

A: Yes, "cleaning" is what was meant. Corrected

Statistical analysis

9. Line 137: ‘our’ should be replaced by ‘the’. Better still, the outcome of interest could be repeated here.

A: Ok, that was corrected.

10. Line 143: What do Authors mean by ‘interactions among our dependents were explored’? Are they referring to (in)dependent variables?

A: Yes, this is a typographical error. Corrected, thank you

Results

11. Line 167: Is that sentence a heading?

A: No, that is the table footnote.

12. Line 173: The caption of Figure 2 needs modification. Proportion of uptake does not read well as ‘uptake’ means the proportion of women who have taken a specific number of IPTp-SP doses.

A: Corrected.

13. Lines 180-183: The interpretations Authors are giving to the figures here are not correct. Authors should interpret the results carefully. Secondly, the degrees of freedom that are reported in Table 1 are very wide; Authors need to state cautiously what the findings are at this stage.

A: We improved the interpretations of our results and reviewed it for clarity.

The degrees of freedom presented here are the one used by the procedure to compute the F statistics in the Rao-Scott and Wald chi-square tests. Actually this F statistics has a distribution with (R−1)(C−1)/(1+a^2) and κ(R−1)(C−1)/(1+a^2) degrees of freedom. The variance estimator (κ) being calculated from the sample design.

I am not sure to understand what you suggest by mentioning they are "very wide".

14. Again, the interpretation of results in lines 201 to 203 is faulty. Authors should kindly look carefully at these. For example, saying that ‘a greater proportion of women having a poor uptake of IPTp-SP among those who received no care (96.6%)’ is not the same as saying that ‘15.3% of the respondents did not receive any ANC with 96.6% of these having a poor uptake of IPTp-SP.’

A: Yes, that was reformulated.

15. Table 3: Women's age category should be 15-25 years according to previous categorization in table 1 and not 0-25 years.

A: Yes, corrected.

16. Line 220: Could Authors consider stating the positive finding instead of the double negative as stated in the sentence? This would state the finding more clearly.

A: Yes, thank you.

Discussion

17. Lines 287 to 291: These are very valid reasons that have been written in this paragraph. Could Authors clarify/simplify the sentences in these lines and possibly add some references? Does 'increase in drug shortage' mean SP stockouts? What does 'an overwhelmed health system from the elevated demand' mean? And does 'dodging of preventive doses because of recent treatment received for frequent infections' mean 'treatment of malaria infections among the pregnant women because they report with signs and symptoms of malaria during ANC rather than giving IPTp presumptively? The policy demands that pregnant women who show signs and symptoms of malaria get tested and those positive treated using ACTs instead of giving IPTp hence the potential reduction in IPTp-SP. This could be elaborated in the discussion.

A: Yes, we meaned "SP stockout". 'An overwhelmed health system from the elevated demand' was a bit of a repeat here, so we removed it. And we reformulated this part to include your valuable comments regarding the policy

- **Reviewer #2:**

Materials and Methods

General: Given that this paper is based on secondary analysis of data collected for another purpose, the details provided on how the original data were cleaned, transformed and manipulated to fit the objectives of the present analysis are missing. Expand description on what figure 1 is showing as well, especially explaining the exclusions of the "unweighted". I would recommend that authors should review some DHS-based papers to see how they have structured the methods sections.

A: Thank you, this part was improved for clarity and details were added regarding manipulation of the data.

1. Lines 109-111: (a) the dependent variable 110 was the number of women who had received less than three doses of IPTp-SP (Poor uptake of 111 ≥3 doses of IPTp-SP). The inclusion of the words in bracket may confuse some readers; consider leaving it out.

A: Yes, thank you. That was reformulated for clarity.

2. Line 111: The use of the word “grooming” seems inappropriate; consider changing to cleaning, preparation for analysis, etc.

A: Yes, we changed that to "cleaning"

Results

3. Tables 1&2: The presentation of “Rao-Scott-F(df1, df2)” and “p-value” columns for categorical variables is a bit confusing; it seems to be linked to the first row of the variable, while it applies to the entire variable. Merge the spaces.

A: Corrected

4. “The 3rd table is not properly labelled as “Table 3”; correct this. In the same table, consider using “REF/Reference” instead of the value “1” in the OR column.

A: Corrected

Discussion

This section can be improved/ strengthen by:

5. Avoid repeating the presentation of results especially the 1st paragraph.

A: Corrected

6. Include a few sentences either at the beginning or at the end describing the strengths of this study, such as use of a large set of databases that is nationally representative, etc.

A: Ok this was done at the beginning of the discussion.

7. Include the limitations of the study; this information is missing.

A: Thank you, we added this section in line 334.

Other comments

Minor language editing required to improve clarity and conform to English grammar.

A: We did some editing to improve clarity.

Dr Dominique K. Guimsop
