# Supplementary material for: Factors associated with the uptake of intermittent preventive treatment for malaria during pregnancy in Cameroon: An analysis of data from the 2018 Cameroon Demographic and Health Survey
Source: PLOS Glob Public Health. 2024 Mar 27;4(3):e0001245. doi: 10.1371/journal.pgph.0001245 (PMC10971583; doi:10.1371/journal.pgph.0001245)
Supplement: Attachment — Submitted filename: Response to reviewers.pdf [file pgph.0001245.s004.pdf]

October 26th 2023

Guimsop Dominique Ken
University of Dschang
BP 67, Dschang Cameroon
dk.guimsop@outlook.com
(+237) 698684550

To,

Dr Orvalho Augusto
Academic Editor
PLOS Global Public Health

Subject: A rebuttal letter

Dear Dr Orvalho Augusto,

Thank you for the opportunity to revise and resubmit our manuscript entitled "Factors associated with the uptake of Intermittent preventive treatment for malaria during pregnancy (IPTp) in Cameroun: an analysis of data from the 2018 Cameroon Demographic Health Survey". We have completed the revisions to the manuscript as suggested by editor and reviewers' helpful comments.

You shall find below under the heading "response to reviewer" an answer to each point raised by the editor and reviewer(s). Each comment is first stated, followed by a paragraph beginning with an "A:" standing for "Answer:".

Response to reviewers

❖ **Reviewer #5:**

- Title for table 1, what are the environmental characteristics presented in the table? Revise the title using appropriate terminology.
  Revise the titles for subsections in the result section, for example the first subsection may be "Demographic Characteristics of Study population".

A: Thank you for your remark. The term "environmental" was deleted from the Table 1 title as it added no relevant information with regard to the quality of the characteristics presented.

Titles for subsections were also revised for coherence:

- the first subsection was entitled "Socio-demographic Characteristics of the Study Population and Comparison with Uptake of IPTp-SP"

- the title for the second subsection was deleted and its content added as a paragraph of the first subsection

- the remaining titles for subsection were corrected to "Obstetrical History of the Study Population and Comparison with Uptake of IPTp-SP" and "Factors associated with Uptake of IPTp-SP" respectively

- ❖ **Reviewer #6:**

ABSTRACT, TITLE

- Title: Factors associated with the uptake of Intermittent preventive treatment for malaria during pregnancy (IPTp) in Cameroon: an analysis of data from the 2018 Cameroon demographic and health survey.

A: Corrected

- The abstract is well-structured, but I have a question regarding the focus of conclusion section. It seems the authors are emphasizing the determinants of optimal uptake of IPTp-SP (≥ 3 doses) rather than the factors associated with poor IPTp-SP uptake, which aligns better with the study's aim. I suggest that they maintain consistency with the study's objectives in presenting the conclusion.

A: As suggested, we replaced the term "determinant" in the background and conclusion sections of the abstract with the term "factors associated"

MAIN MANUSCRIPT CONTENTS

1. Background

In lines 73-76, the authors have mentioned the prevalence range of optimal IPTp-SP uptake in relation to socio-demographic characteristics, ANC (Antenatal Care) utilization, and study design in Cameroon. They also pointed out that there have been few studies conducted in Cameroon that explored the association of socio-demographic factors and “antennal parameter” (what is the antennal?) with IPTp-SP uptake (as seen in lines 77-78). Given this information, I am not entirely convinced about the novel contribution of this present study to the literature on IPTp-SP uptake in Cameroon. The authors have not

thoroughly critiqued previous studies conducted in Cameroon to establish the specific gap that this study aims to address.

A: We revised our gap and justification in line 80-86 for clarification:

"Few small-scale studies were conducted that explored socio-demographic and antenatal parameters that may influence the uptake of IPTp-SP in Cameroon. The range of investigated factors was non-exhaustive and we observe some mitigated results attributable to the use of unharmonized procedures and varying study designs. Because of the potential value of such findings in informing policymakers about strategies to improve the effectiveness of existing approaches or propose new ones we seek to contribute to understanding of the mechanisms behind poor uptake of IPTp-SP."

2. Background

2.1. Statistical analysis

- In line 161, the authors have stated that all independent variables were dichotomized to assess their association with IPTp uptake. It is important to note that logistic regression can be applied to independent variables with various levels of measurement, not just dichotomous variables. Dichotomizing variables is a well-known practice, but it can lead to a loss of information and potentially introduce residual confounding. Therefore, I have reservations about the rationale behind this extensive dichotomization.

A: Thank you for your comment. We dichotomized the variables before running bivariate and multivariate regression analysis. Our rationale is that, as per usual in the medical field, disease or exposure is defined by the position of the value of a subject characteristic with regard to a cut-off point. On that basis, dichotomization allows for ease of interpretation and formulation of recommendations. It is however important to note, as you remarked, that this practice can often lead to loss of information and residual confounding.

To limit that effect, we revised the manuscript the following way: continuous variables (Age and Area's malaria prevalence) in our database were only stratified by more than 5 categories before being added to the regression analysis [1, 2]; while discrete and categorical variables were kept dichotomized around our cut-off value or characteristic of interest.

We found that the variable "Age", as a categorical variable, did not met the criteria to be included in the multivariate logistic regression model and was removed from "Table 3",

along with "Length of stay in the place of residence" and " Current occupation", and "Illiterate"

2.2. Statistical model used

- In lines 160-161, the manuscript mentions the use of bivariate logistic regression. It is not clear whether this analysis is within a multilevel modelling (MLM) framework or standard logistic regression. If it's standard logistic regression, an explanation for this choice would be beneficial.

  Additionally, in line 172, it is indicated that MLM was applied for each covariate. The rationale for employing standard regression in the initial stage and MLM in the second stage should be clarified.

  A: As specified in line 156 under the Data Management section:

  "The sampling design of the survey was accounted for by using the "Complex Samples" package of IBM SPSS for the analyses."

  So, all analysis were made within the MLM framework. However, to add clarity, we rephrased and specified in line 168 that this analysis was done "within the multilevel modelling framework".

- Furthermore, in lines 188-189, the manuscript states that the analysis accounted for the survey design. If multilevel modelling analysis incorporated weights, it would be valuable to provide a brief description of the methods used in this regard.

  A: We revised the Data Management section and added a sentence that briefly describes the methods used to incorporate weights to our MLM analysis, we added in line 161:

  "This package allows for specification of the sample scheme parameters, including stratification, clustering, weighing, and inclusion probabilities, to accurately estimate standard errors. Sample weights were provided by the "weight" variable from the database."

- In line 192, the manuscript mentions the fitting of interaction terms. It would be beneficial to clarify whether these interaction terms were pre-planned as part of the study's hypothesis and design or if they were determined through a trial-and-error process during the analysis. Understanding the rationale behind the inclusion of interaction terms can provide useful insight.

  A: Thank you for your comment. We revised this sentence to:

"Substantive cross-level interactions among our independent variables were then explored through a trial-and-error process to understand the effect of community-level characteristics on the individual-level associations"

3. Results

- In the methods section, the authors state that all independent variables were dichotomized (line 161). However, upon reviewing Table 1, it is evident that not all variables are dichotomized. The methods section should be revised for accuracy and consistency.

  A: As specified in line 160, we first used the Chi-square test to compare the distribution of the independent variables between the two levels of our dependent variable. This was done using independents categorical variables in their initial (non-dichotomized) form as a mean to explore their relationship with the dependent. It is these results that are presented in Table 1 & 2.

  Variables were subsequently dichotomized and included into bivariate and multivariate regression analysis (Table 3).

- Additionally, in Table 1, it would be beneficial for the authors to include a footnote indicating that the sample sizes presented are weighted, as this is important information for the readers to understand.

  A: This concern was already addressed under the Data Management section by specifying that: "Unless otherwise specified, only weighted data were presented in the results."

- Furthermore, in line 233, the authors claim a statistically significant difference between higher education and secondary education, even though there are four levels of education mentioned. It is unclear which specific level(s) of education are driving this statistical significance. In my opinion, it is difficult for the authors to specify the particular education levels for which a statistically significant difference was found. The way conclusions are drawn involving more than two levels of variable measures should be revised in this manuscript.

  A: Thank you. We revised the result section accordingly.

  Necessary changes were made:

- in line 146: "we observe a significant variation of the uptake of the recommended 3 doses of IPTp-SP across levels of education ($p < 0.001$), women who attended higher levels of education were more likely to take the ≥3 doses."

- and in line 268: "There was a significant association between the qualification of the ANC attendance and uptake of IPTp-SP ($p < 0.001$)"

- Does the Table 3 include the MLM bivariable logistic regression or the standard logistic regression?

A: Please see # 2.2 above. No results presented in this manuscript were obtained from standard logistic regression.

4. Discussion

- I recommend that the statements found in lines 292-299 be incorporated into the 'strengths of the study' section, as they seem to highlight specific strengths or advantages of the research.

A: Following the changes in the result section, we revised our discussion for coherence. We also changed the title "Limitations" to "Strength and limitations" (line 367) and inserted the statements found in lines 292-299 under that section as recommended.

- Results in lines 342-343 should be interpreted with caution because the p-value is weak as evidence with 95% confidence.

A: We highlighted this concern by adding the phrase "Despite a weak statistical significance," at the beginning of the sentence.

Thank you for your time and consideration with reviewing this manuscript.

Sincerely,

Dominique Guimsop MD, MPH (on behalf of the authorship team)

References

1. Brenner H, Blettner M. Controlling for continuous confounders in epidemiologic research. Epidemiology. 1997;8(4):429-34.
2. Groenwold RH, Klungel OH, Altman DG, van der Graaf Y, Hoes AW, Moons KG. Adjustment for continuous confounders: An example of how to prevent residual confounding. CMAJ. 2013;185(5):401-6. 10.1503/cmaj.120592.
