# Supplementary material for: Factors associated with the uptake of intermittent preventive treatment for malaria during pregnancy in Cameroon: An analysis of data from the 2018 Cameroon Demographic and Health Survey
Source: PLOS Glob Public Health. 2024 Mar 27;4(3):e0001245. doi: 10.1371/journal.pgph.0001245 (PMC10971583; doi:10.1371/journal.pgph.0001245)
Supplement: Attachment — Submitted filename: Response to reviewers_R5.pdf [file pgph.0001245.s005.pdf]

January 28th 2024

**Guimsop Dominique Ken**
**University of Dschang**
BP 67, Dschang Cameroon
dk.guimsop@outlook.com
(+237) 698684550

To,

**Dr David Musoke**
Academic Editor
PLOS Global Public Health

Subject: A rebuttal letter

**Dear Dr David,**

Thank you for the opportunity to revise and resubmit our manuscript entitled "Factors associated with the uptake of intermittent preventive treatment for malaria during pregnancy in Cameroon: An analysis of data from the 2018 Cameroon Demographic and Health Survey". We have completed the revisions to the manuscript as suggested by editor and reviewers' helpful comments.

You shall find below under the heading "response to reviewer" an answer to each point raised by the editor and reviewer(s). Each comment is first stated, followed by a paragraph beginning with an "A:" standing for "Answer:".

Response to reviewers

❖ **Reviewer #5:**

- Line 278 – 282: "This indicates ......... Specifically, ......... ". These statements should be moved to discussion section.
  I believe there is still a need for improvement in terms of language. Hence, I recommend that an English editor revise the manuscript.

**A**: Thank you so much.

These statements were deleted at once, since they were already repeated in the discussion section.

An English editor revised the manuscript as suggested, and corrections were made.

❖ **Reviewer #6:**

**ABSTRACT, TITLE**

- The abstract is well-structured, but I have a question regarding the focus of conclusion section. It seems the authors are emphasizing the determinants of optimal uptake of IPTp-SP (≥ 3 doses) rather than the factors associated with poor IPTp-SP uptake, which aligns better with the study's aim. I suggest that they maintain consistency with the study's objectives in presenting the conclusion.

  Your response on the above suggestion shows that you did not understand what I meant. It is not about the mere word "determinant" but your aim and conclusion are not matching. You aimed to investigate the distribution and factors associated with poor uptake of IPTp-SP… but your conclusion is highlighting the optimal uptake of IPTp-SP.

**A:** We reformulated the first statement of the conclusion to align with the study objectives. Sorry I misunderstood you in the previous revision, I hope I got your point this time.

**MAIN MANUSCRIPT CONTENTS**

**1. Background**

In lines 73-76, the authors have mentioned the prevalence range of optimal IPTp-SP uptake in relation to socio-demographic characteristics, ANC (Antenatal Care) utilization, and study design in Cameroon. They also pointed out that there have been few studies conducted in Cameroon that explored the association of socio-demographic factors and "antennal parameter" (what is the antennal?) with IPTp-SP uptake (as seen in lines 77-78). Given this information, I am not entirely convinced about the novel contribution of this present study to the literature on IPTp-SP uptake in Cameroon. The authors have not thoroughly critiqued previous studies conducted in Cameroon to establish the specific gap that this study aims to address.

> I remain unconvinced by the new justification. You have cited only two studies (DHS and Diengou et al, 2020) as your evidence, yet there are numerous studies conducted in Cameroon on this subject. Perhaps it would have been more accurate to mention the limited literature available on this topic from a nationally representative sample, which the Demographic and Health Survey provides.

**A:** Thank you, Dr. We added this statement to the justification in line 76:

"However, there is limited literature available, from a nationally representative sample, which have explored socio-demographic and antenatal parameters that may influence the uptake of IPTp-SP in Cameroon."

We also corrected in-text citation in line 74 to refer to the 6 articles we consulted which evaluated IPTp-SP rates in Cameroon.

**2. Results**

- In the methods section, the authors state that all independent variables were dichotomized (line 161). However, upon reviewing Table 1, it is evident that not all variables are dichotomized. The methods section should be revised for accuracy and consistency.

  Based on your response, why can you not add the statement that you used to respond to my query? I suggest that you include the statement that you used to respond to my query.

**A:** We corrected the statistical analysis section in line 154-159 to include the statement as suggested. It now reads:

"The Rao-Scott chi-square test was used to compare the distribution of the independent variables between the two levels of our dependent variable. This analysis tested for differences within groups while adjusting for sample design using an F statistic and was done using independents categorical variables in their initial (non-dichotomized) form (Table 1 & 2). All independent variables were subsequently dichotomized and bivariate logistic regression was used within the multilevel modelling framework to measure the independent variables' association with the uptake of fewer than three doses of IPTp-SP (Table 3)."

- Additionally, in Table 1, it would be beneficial for the authors to include a footnote indicating that the sample sizes presented are weighted, as this is important information for the readers to understand.

  Information presented in Tables should provide comprehensive details without relying on references to other parts of the document. It is concerning to find a table containing information that depends on definitions found in specific sections of the document. Tables should be self-contained and independent. I am not impressed with your response.

**A:** Thank you so much.

A footnote was added bellow every tables indicating that: "all presented sample sizes and frequencies are weighted"

Thank you for your time and consideration with reviewing this manuscript.

Sincerely,

**Dominique Guimsop MD, MPH** (on behalf of the authorship team)
