# Supplementary material for: Factors associated with the uptake of intermittent preventive treatment for malaria during pregnancy in Cameroon: An analysis of data from the 2018 Cameroon Demographic and Health Survey
Source: PLOS Glob Public Health. 2024 Mar 27;4(3):e0001245. doi: 10.1371/journal.pgph.0001245 (PMC10971583; doi:10.1371/journal.pgph.0001245)
Supplement: Attachment — Submitted filename: Response to reviewers.docx [file pgph.0001245.s001.docx]

Guimsop Dominique Ken

**University of Dschang**

January 12^th^ 2023

BP 67, Dschang Cameroon

[dk.guimsop@outlook.com](mailto:dk.guimsop@outlook.com)

(+237) 698684550

To,

**Dr Catherine Kyobutungi**

Editor-in-Chief

PLOS Global Public Health

Subject: A rebuttal letter

**Dear Dr Kyobutungi**,

I am pleased to submit the revised version of my original manuscript after consideration of the how pertinent comments of the reviewers. I wish to thank you and the team for the meticulous work, and hope to hear from you soon.

You shall find below under the heading “response to reviewer” an answer to each point raised by the editor and reviewer(s). Each comment is first stated, followed by a paragraph beginning with an “A:” standing for “Answer:”.

Response to reviewers

- **Reviewer #1:**

- Lines 29-30: you mentioned, "Frequencies of mother exposed to less than 3 doses of IPTp-SP were estimated with a 95% CI". However, I went through all the results but did not see where you presented these frequencies with their confidence intervals

**A:** Our apology, latter in the course of the work we opted for keeping confidence intervals only for Odd ratios in order to render the work less bulky except the one for our main finding (wich you can see in the abstract - line 36). however we omitted to correct this sentence. It is now corrected

- Line 32: you did not specify the statistical model used to estimate the crude and adjusted odd ratios, but this should be mentioned in the Abstract's method section.

**A:** Addressed. We used multilevel binary logistic regression.

- Line 45: This is the conclusion and cannot be started with the word "Therefore." It would be best if you replaced it with an appropriate term for a conclusion like to sum up, In conclusion, or even to conclude.

**A:** Addressed. Thank you

- Line 47: You did within comparison for the Sahelian region, but it would be more interesting to compare the sahelians to the others regions and not within the same region.

**A:** What we actually did was compare women from the sahelians to women from all other regions, wich gave us the obtained result (line 39)

- Line 66: can you provide more data on the overall range of the coverage in terms of the minimum and the maximum?

**A:** Yes, the range was added.

- Line 76 – 77: You mentioned that the gap was to understand the limitation of the health care system in achieving its objectives; meanwhile, in this current study, the main targets was to determine the coverage and understand the socio-demographics parameters that affect the uptake of IPTp-SP in Cameroon. In other words, you did not collect any healthcare system parameters in your study. Therefore, there is a need to re-formulate this sentence.

**A:** Ok, we reformulated the sentence

- Line 89: can you elaborate more on the two-stage sampling process, I mean, the primary sampling unit, the secondary sampling unit, and so on?

**A:** Yes, we added some details on the methodology ( line 91-98)

- Line 95: There is a missing word cause you mentioned "A living child less than at the day the selected household is visited. I think the missing word is five. Cause you mean less than 5 years old?

**A:** Yes thank you, we corrected it.

- Lines 110: The definition of the dependent variables is not clear. The dependent variable is the uptake of IPTp-SP and comprises two categories, < 3 doses and ≥ 3 doses. Therefore, there is a need to rewrite this sentence.

**A:** Yes, we did rewrite it.

- Line 122, line 128: you mentioned that you used the complex sample's functionality in SPSS due to the structure of your data. Does this account for the cluster sampling aspect of your study? In other words, does this complex sample functionality account for mixed effects models?

**A:** the "Complexe samples" functionality within SPSS allows for different procedures. We used the Complex Samples Logistic Regression procedure to analyze our data. This procedure performs multilevel binary logistic regression analysis for samples drawn by complex sampling designs (including clustering and stratification). It builds a model that accounts for the design effect, estimating main-effects, interaction effects and nested terms as appropriate.

As mentioned (Line 134), empirically relevant interactions were tested and only those found statistically significant were included in the final model. No nested term was added to the model

- Line 130: You mentioned, "Dependent that had an association with the outcome." Dependent, outcome, and response variables are the same. Therefore, there is a need to correct this. I think you mean independent, which had an association with the outcome.

A:Thank you. that was corrected

- Line 157: Table 1: I prefer the Mother's age (years) instead of Age groups (years). Also, it is better to present the proportions row-wise instead of column-wise. In addition, you should compare the proportions between levels instead of within levels.

**A:** We changed "Age groups (Years)" to "Women's age" for consistency since "women" is the term I use throughout the document.

we provided between level comparisons, as suggested, and presented column-wise proportions.

- Lines 169: You started describing the data without indicating in which Tables we can find these data. In the first sentence of this paragraph, you should introduce the Table and then follow with the data description.

**A:** this was corrected.

- Line 180: You should provide a between-regions comparison and not within regions.

**A:** we provided between level comparisons, as suggested.

- Line 184: You should first introduce the tables, and the proportion should be presented row-wise instead of column-wise as you did.

**A:**Thank you. we added a sentence to introduce the table.

We also changed column-wise for row-wise proportion in our tables (execpt for the Total's column) and indicated it in our header rows.

- Line 186: You mentioned parity below 6, but in the Table, we only have parity ≤ 5.

**A:** thank you. this actually represent the same information. however, I corrected the text match the label in the table.

- Line 217: You omitted to add Table 3 before the title "Determinants of the uptake ....

**A:** corrected

- **Reviewer #2:**

Major comment

The authors should find a native English speaker to assist correcting the language.

**A:** Thank you. We did try to correct the language.We also were assisted by a native neglish speaker on that matter.

Minor comments

- Line 21: Change …..increasing mother and children mortality…….to…..increasing pregnant women/mothers and children mortality…..

**A:** Done

- Line 23: WHO is mentioned for the first time, thus it should be written in full followed by the abbreviations in brackets, thereafter, you can continue using the abbreviations.

**A:**Yes, we missed it. Thank you

- Line 24: Present study aimed…and not…aims.

**A:** Corrected

- Line 27: Change…administered in face to face…to….administered face to face…or ,..administered directly.

**A:** Done

- Line 28:….one child under 5…what is the unit of measurement, years or months?

**A:** It is "Years" we corrected that.

- Line 30: …..A case-control design….Can you explain how this was a case control design study?

**A:** we didn't actually call it a case control design study. We simply pointed out that we applied the case-control design methodology (from definition of our outcome -ie cases vs controls-... to estimation of odd ratios) to our database to test the associations.

- Line 45: The conclusion is not clear, re-write to increase its clarity.

**A:** Ok, we corrected the sentences to make it clear.

- Line 65: Despite implemented…..to….Despite the implementation of…..

- Line 68: …..factors as level…..to….factors such as /or including…….

**A:**Both Corrected

- Line 70: The sentence is not clear, re-write to increase its clarity…e.g.,…the Ministry of Health adopted IPTp using chloroquine since 2002….

**A:** Yes thanks, we corrected it.

- Line 71: ANC….write in full, followed by the abbreviations in brackets, thereafter, you can continue using the abbreviation.

**A:** Done

- Line 72: The sentence is not clear, rephrase.

**A:** Rephrased

- Line 95: ……biological mothers of a living child aged less than….less than what? Also, ……household is visited…….change to…..household was visited.

- Line 100: ….a under 5 years old….to…an under 5 years old….

- Line 102: …..and those did not have…..to….and those who did not have…..

**A:** All corrected.

- Line 111: …..stay in the place of residence……change to……residents of the catchment area…..

**A:** Thank you for your proposition, but it does not seem to catch the idea.

what we were referring to here was actually the duration of time that the individual has spent in his current residence. So we corrected it to "length of stay"

- Line 112:Why have both “woman’s highest education level” and “literacy”? These two assess the same thing.

**A:** For the sake demographic health surveys (DHS), these 2 variables though consitently related were defined separately. "Literacy" here reffers solely to the ability to read and write, independently of the level reached in school.

- Line 115: ANC highest attendant….this is not clear. I believe you meant the cadres of the attendants i.e., nurse, doctor, etc. Find a good way to express it to increase clarity.

**A:** Ok, this was changed to "cadres of the attendants".

- Line 130-131: Dependents that…..were selected for multivariate logistic regression model. This sentence is not clear, rephrase to increase clarity.

**A:** rephrased to add clarity

- Line 151:……instead of (48.1%, n=2657)…..change to…..48.1% (2657/5528). Change throughout the results section.

**A:** Ok, we did that.

- Line 151-52:….with almost half of aged…to….with almost half of them aged…., also add a comma between “old” and “and”.

**A:** Yes, thanks. We corrected both of these.

- Line 152: ….., and 44.5% having….to…and 44.5% of them having…..

**A:** Done

- Line 153: About half (52.6% (2911/5528)…..this is more than half, not about half. Also change….and they seem to….to……and they seemed to….

**A:** Ok, we did it.

- Table 1: -Why the age group of 40-49 has a range of 9 years while all other age groups have a range of 4 years?

**A:** Yes, we decided to join the 40-44 and 45-49 groups because the latter didnt have much occurences

- Stay in the place of residence…to.. reside/or resident of the catchment area.

**A:** We changes "Stay in the place of residence" to "Lenght of stay in the place of residence"

- Highest education level…..to level of education.

**A:** Corrected

- Illiterate is part of the level of education, so it should be combined with no formal education.

**A:** Note really: one may have learned to read out of school, also one may have attended some level of education and yet be unable to read.

- Current occupation…….I was expecting to see different groups of occupation i.e., peasants, petty traders, government employee etc.

**A:** The term "current occupation" relates to wether or not the participant had a job at the moment of the study.

- Line 167: Subheading….Sociodemographic characteristics of participants and uptake of <3 doses of IPTp-SP……refers to the same things as the subheading…..Determinants of the uptake of less than 3 doses of IPTp-SP. These two sections should be combined and streamlined to avoid repetition.

**A:** Please I am not sure to understand:

Under the subheading "Sociodemographic characteristics of participants and uptake of ≥3 doses of IPTp-SP……" you have descriptives statistics of sociodemographic characteristics and the results of univariate analyses.

Under the subheading "Determinants of the uptake of less than 3 doses of IPTp-SP" you have the results of multivariate analyses.

So, there is no repetition here and joining the will result in a very long section.

- Line 227: Since 2004 in Cameroon……to…In Cameroon, since 2004 IPTp-SP…..

- Line 275: ….service overwhelming……to….health system overwhelmed…..

**A:** Both corrected. **Many Thanks!**
