# Supplementary material for: Factors associated with the uptake of intermittent preventive treatment for malaria during pregnancy in Cameroon: An analysis of data from the 2018 Cameroon Demographic and Health Survey
Source: PLOS Glob Public Health. 2024 Mar 27;4(3):e0001245. doi: 10.1371/journal.pgph.0001245 (PMC10971583; doi:10.1371/journal.pgph.0001245)
Supplement: Attachment — Submitted filename: Response to reviewers.docx [file pgph.0001245.s003.docx]

Guimsop Dominique Ken

**University of Dschang**

July 31^th^ 2023

BP 67, Dschang Cameroon

[dk.guimsop@outlook.com](mailto:dk.guimsop@outlook.com)

(+237) 698684550

To,

**Dr Orvalho Augusto**

Academic Editor

PLOS Global Public Health

Subject: A rebuttal letter

**Dear Dr Orvalho Augusto**,

Thank you for the opportunity to revise and resubmit our manuscript entitled “Factors associated with the uptake of Intermittent preventive treatment for malaria during pregnancy (IPTp) in Cameroun: an analysis of data from the 2018 Cameroon Demographic Health Survey” We have completed the revisions to the manuscript as suggested by editor and reviewers’ helpful comments.

You shall find below under the heading “response to reviewer” an answer to each point raised by the editor and reviewer(s). Each comment is first stated, followed by a paragraph beginning with an “A:” standing for “Answer:”.

Response to reviewers

- **Additional editor comment:**

**General**

**1.** Somewhere in the abstract, it is said that the author conducted a multilevel analysis. However, we have no details of such a procedure in the methods, neither the tables (eg Table 3) with associations suggest such a procedure has ever been used here. Please add more details in the statistical analysis of what was done to perform this analysis.

**A:** We have made revisions in the manuscript to provide details on how we performed the multilevel analysis. Details on the model and how it was made can be found in the *Methods section, Statistical analysis, lines 168 to 192*:

**"**This The two-level (multilevel) binary logistic regression model was run to examine the association (measure of effect) between the outcome and each independent variable while controlling the effects of the others. It estimated the adjusted odds ratio (exp(ꞵ)) accounting for the unobserved characteristics at cluster or community level (second level) during the data collection in addition to the women individual characteristics (first level). Its notation is as follows:

$$\log\left( \frac{\pi_{ij}}{1-\pi_{ij}} \right)=\beta_{0}+\beta_{1}x_{1ij}+\beta_{2}x_{2j}+u_{j}$$

where, uj ~N(0, σu2) and πij = the probability of an event occurring for the i level 1 unit in the j level 2 unit; β0 is the log-odds that y = 1 when x = 0 and u = 0; β1 is effect on log-odds of 1-unit increase in x for individuals in same group; uj is the effect of being in group j on the log-odds that y = 1, also known as a level 2 residual; σu2 is the level 2 (residual) variance, or the between-group variance in the log-odds that y = 1 after accounting for x; $x_{1ij}$ is a generic level one nested within level 2 independent variable; $x_{2j}$ indicates a level two independent variable.

The design effect (The ratio of the variance of the estimate to the variance obtained by assuming that the sample is a simple random sample) was first checked on intercept-only model and was found to be 128.01, indicating a significant effect of specifying the complex design. Random intercepts were retained. The full model was then made by entering all selected independent variables simultaneously.

Collinearity was checked using Variance inflation factors (VIFs) calculation. Collinearity was considered high for covariates with a VIF greater than 4, which indicates a twofold increase in the standard error of a regression coefficient, in presence of collinearity.

Finally, substantive cross-level interactions among our independents were then explored, and only one was found significant, which was retained in the model. The final model had and Nagelkerke R2 of 0.201. The level of significance used was 5% (0.05), two-tailed at 95% confidence interval (CI).**"**

Moreover, we parted our independent variables in *Table 3* into 3 categories: Individual-level characteristics, community-level characteristics, and interaction terms; for the reader to easily identify the level of collection of each variable

**2.** The abstract is too long. Please add the subsections (background, methods, results, and conclusions to help the reader follow).

**A:** The corresponding subsections titles were added to the abstract.

- **Reviewer #5:**

**1.** According to the manuscript, the objective was to determine factors contributing to the low uptake of IPTp. But the authors presented factors for both high and low uptake of IPTp in results, discussion and conclusion sections; this may confuse readers of the article. I recommend the authors to put their focus on the factors for low uptake of IPTp.

**A:** Thank you for this suggestion. We revised our analyses and reset the reference categories of the concerned dependent variables such that we can emphasize on factors for low uptake of IPTP only. The results, discussion and conclusion sections were corrected accordingly.

**2.** The authors should consider revising the analysis (especially the logistic regression). In most cases, the multivariate logistic regression analysis includes independent variables that have attained a certain level of significance (usually a pvalue of less than 0.2) during the univariate logistic regression analysis. This will enable authors to (1) consider only significant variables and (2) adjust for confounders.

**A:** thank you for this comment. This is exactly what was done. There was a typo at line 160 where it was written p<0.05 instead of p<0.20 and we corrected the sentence to **"**Independent variables that were associated with the outcome at significance level of p<0.20 were selected as candidates for the main effects in the multilevel binary logistic regression model**"**.

Most of our independent variables responded to this criterion and were thus added to the multivariate model. As specified in the *Methods section, Statistical analysis, line 161 to 168*:

**"**Only two of our independent variables did not attain that level of significance on bivariate regression analysis, namely “Length of stay in the place of residence” and “Current occupation” and were consequently not included in the multilevel model. Collinearity was checked using Variance inflation factors (VIFs) calculation. Collinearity was considered high for covariates with a VIF greater than 4, which indicates a twofold increase in the standard error of a regression coefficient, in presence of collinearity. The variable "Illiteracy" was removed from the model because of suspected collinearity with "Level of education"**"**.

Moreover, we modified Table 3 to allow for the presentation of the p-values from the Univariate analysis.

**3.** Authors should rewrite the references appropriately and consistently both in text (using brackets instead of parentheses) and the reference list, e.g., reference number 2. Authors should adhere to the journal guidelines for writing references (Vancouver style).

**A:** Citations (reference in text) were checked and were not sure what the reviewer wanted corrected here. Please could the reviewer specify?

Reference number 2 was corrected in the reference list: Authors and journal abbreviations were cited according to Vancouver style. The whole reference list was similarly revised to ensure that it reference meets journal requirements, and made the necessary corrections.

**4.** To improve the manuscript's language, the authors should send it to a professional English editor.

**A:** We worked with an editor to improve the manuscript language. The entire manuscript was revised to conform to English grammar, punctuation, spelling and consistency to the North America English dialect and changes were made to improve clarity.

Thank you for your time and consideration with reviewing this manuscript.

Sincerely,

Dominique Guimsop MD, MPH (on behalf of the authorship team)
